# The Modified Harrington Procedure for Metastatic Peri-Acetabular Bone Lesion Using a Novel Highly Porous Titanium Revision Shell with Long Lever Arm Screw

**DOI:** 10.3390/medicina60071047

**Published:** 2024-06-26

**Authors:** Sven Frieler, Carsten Gebert, Yannik Hanusrichter, Periklis Godolias, Martin Wessling

**Affiliations:** 1 Department of Tumour Orthopaedics and Revision Arthroplasty, Orthopaedic Hospital Volmarstein, 58300 Wetter, Germany; 2 Department of Trauma and Orthopaedic Surgery, BG University Hospital Bergmannsheil, Ruhr University Bochum, 44801 Bochum, Germany; 3 Seattle Science Foundation, Seattle, WA 98122, USA; 4 Department of Orthopedics and Tumor Orthopedics, Muenster University Hospital, 48149 Muenster, Germany; 5 Center for Musculoskeletal Surgery, University Hospital of Essen, 45147 Essen, Germany; 6 Department of Orthopedics and Trauma Surgery, St. Josef Hospital Essen-Werden, 45239 Essen, Germany

**Keywords:** peri-acetabular metastases, modified Harrington procedure, Harrington procedure, MUTARS^®^ PRS^®^, osseous ingrowth, immediate full weight-bearing

## Abstract

*Background and Objectives*: Peri-acetabular metastases often lead to significant pain and functional impairment. Surgical interventions, including the Harrington procedure, aim to address these challenges. This study evaluates a modified Harrington procedure using the MUTARS^®^ PRS^®^ (Pelvic Revision Shell) with an 8 mm fixation screw for severe acetabular defects resulting from metastatic lesions. *Materials and Methods*: Retrospective analysis of 12 patients treated between January 2020 and December 2023 was conducted. The procedure involved using the novel MUTARS^®^ PRS^®^ with an 8 mm in diameter dome screw (length 70–100 mm). Outcome measures included implant positioning changes, complication rates, functional outcomes, implant longevity, and patient survival. Radiological assessments were performed postoperatively, with follow-ups at 3, 6, 12 months, and annually thereafter. *Results*: Average follow-up was 15 ± 11 months, with 67% patient survival at 1 year and 44% at 2 years. Implant survivorship remained 100%. Harris Hip Score improved significantly from 37 ± 22 preoperatively to 75 ± 15 at the last follow-up. No revisions involving implant components were reported. Complications occurred in 5 of 12 patients. Overall, PRS^®^ demonstrates effective osseous ingrowth, high primary stability, immediate full weight-bearing, and low complication rates. *Conclusions*: PRS^®^ integrates facilitating osseous ingrowth for preferable long-term outcomes, while efficiently transmitting the weight-bearing load to the intact aspect of the pelvis using a long 8 mm lever screw, enhancing the primary stability of the construct. It proves to be an effective and reproducible technique for managing destructive metastatic lesions of the acetabulum and peri-acetabular region, even in irradiated bone.

## 1. Introduction

The pelvis emerges as a frequently impacted region in patients with bony metastases. Substantial pain and impairment are subsequent to destructive peri-acetabular metastases exposed to mechanical forces of the hip. Treatment options include non-operative approaches such as systemic chemotherapy, immunotherapy, local radiation, hormonal treatment, and bone-modification agents as well as surgical treatment with reconstruction of the pelvis. Decision making is based on the functional status, prognosis for survival, comorbidities, and the response to non-operative treatment [1].

However, the establishment of a widely accepted gold standard in surgical procedures has eluded consensus. The reconstruction of peri-acetabular bone lesions remains challenging. General objectives include removal of the evident tumor with intralesional curettage and reconstruction of the peri-acetabular defect, achieving stable fixation to ensure implant survival for the remaining expected lifetime, absence of pain, and immediate full weight-bearing postoperatively.

In 1981, one of the most profound surgical concepts in managing peri-acetabular metastatic disease was published—the Harrington procedure. It is based on the idea of transmitting the weight-bearing load of the weakened acetabulum to the intact proximal aspect of the pelvis [2]. Since then, various modifications have been published in order to improve surgical treatment and functional outcomes. Vielgut et al. used two to three threaded pins with cement augmentation to restore bony continuity and achieve stable fixation for total hip arthroplasty (THA) [3]. Kask et al. based the reconstruction on a restoration reinforcement ring in combination with cement augmentation of bone defects, cemented cups, and cannulated fixation screws [4]. In 2020, Houdek et al. utilized highly porous tantalum acetabular components and augments successfully with fewer complications compared to cemented Harrington-style techniques [5].

The primary objective of the study was to evaluate the feasibility and safety of a modified Harrington procedure combining the advantages of different fixation philosophies previously described. The concept is based on a novel generation of off-the-shelf (OTS) highly porous titanium constructs supported by the original concept idea of transmitting the weight-bearing load to the intact aspect of the pelvis utilizing a long lever screw. The PRS^®^ (Pelvic Revision Shell) aided by an 8 mm fixation screw (Implantcast GmbH, Buxtehude, Germany) enables full weight-bearing in patients with severe acetabular defects due to metastatic lesions. Outcome measures included changes in implant positioning, complication rates, functional outcome, implant longevity, and patient survival.

## 2. Materials and Methods

Medical records for analysis were obtained from a high-volume tertiary referral orthopedic center’s prospectively maintained hospital database. Patient data were collected prospectively and evaluated retrospectively. Approval for the study was obtained from the Institutional Review Board (IRB reference number 2023-624-f-S). Solely patients with a minimum follow-up (FU) of 6 months were included. Overall, peri-acetabular metastasis in 12 patients treated surgically with the modified Harrington procedure was included in the analysis between January 2020 and December 2023 (Table 1). Primary malignancies were not in the scope of this study, given the varied treatment approaches associated with them.

The decision-making process for surgery involved the patient and a senior consultant, respecting the entire history of disease and the department responsible for treating the primary tumor (chemo- and radiotherapy) and was based on pain relief and restoring the ability to ambulate. No other predefined criteria were used.

Preoperative radiological assessment was performed by plain radiographs, CT, and MRI. Metastatic lesions were classified according to Harrington (Harrington 1981).

### 2.1. Implant Features

MUTARS^®^ PRS^®^ (**P**elvic **R**evision **S**hell; Implantcast GmbH, Buxtehude, Germany) is a novel all-titanium cementless hemisphere flattened at the pole reconstruction shell used in extensive cavitary or segmental acetabular defects up to Paprosky type 3b ± discontinuity. A conventional cemented acetabular cup combination with a dual mobility liner was placed in the acetabular construct. PRS^®^ features three holes for an 8 mm cancellous bone screw, as well as nine holes for 6.5 mm cancellous bone screws.

### 2.2. Surgical Procedure

**Figure 1:**
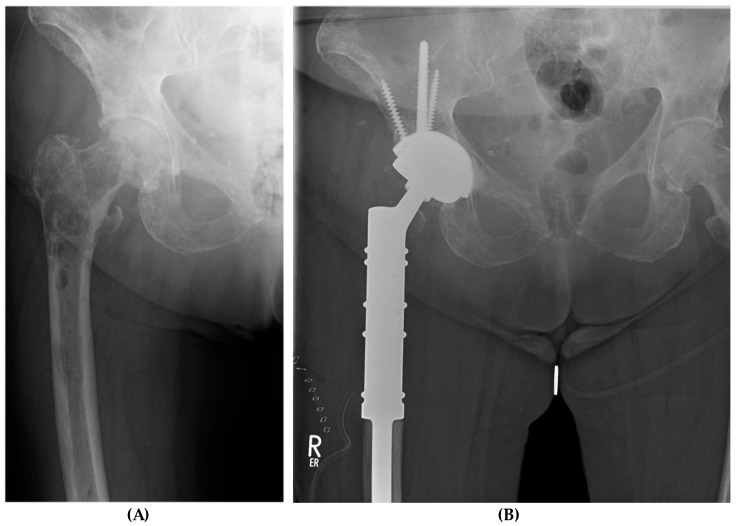
Case report of a 75-year-old female (#12); metastatic breast cancer. (**A**) Local progress of metastatic destruction femoral and peri-acetabular after radiation. Immobilizing and therapy-resistant pain. (**B**) Radiograph post-surgery; implant system: MUTARS^®^ PRS^®^ (Pelvic Revision Shell; Implantcast GmbH, Buxtehude, Germany); MUTARS^®^ Proximal Femoral Replacement (Implantcast GmbH, Buxtehude, Germany). Patient survival at 36 months, absence of pain; patient regularly uses a cane for longer walking distances.

All procedures were performed by orthopedic oncologists. Patients were placed under general anesthesia. A preoperative prophylactic single-shot antibiotic was used. Lateral or posterior standard approaches were used for hip dislocation and resection of the neck, subsequently allowing for extended visualization of the acetabulum and ensuring curettage of peri-acetabular metastases. Metal augments were used to address defects in the weight-bearing zone. Lacunar defects are traditionally addressed using cement augmentation. For larger defects, additional reinforcement of the defect zone can be achieved using Steinmann pins or screw placement. PRS^®^ is loosely placed in the augmented defect zone while a positioning guidewire followed by an 8 mm cannulated dome screw are inserted along the iliolumbar bar, but final fixation is avoided. Subsequently, a partial press-fit fixation with cement reinforcement in the area of the defect is achieved with conventional techniques and further primary stability is ensured using additional 6.5 mm fixation screws. Prior to the placement of a cemented tripolar revision cup, the 8 mm screw is tightened while the cement for augmentation and reinforcement sets. Different cemented and uncemented femoral components were used (Figure 1).

### 2.3. Clinical and Radiographic Assessments

To ensure accurate placement of the 8 mm dome screw intra-operatively, the authors prefer the use of fluoroscopy, and a CT scan was performed postoperatively on the first days following surgery. All patients were mobilized postoperatively, allowing immediate full weight-bearing.

Patients were evaluated at the outpatient clinic at 3, 6, and 12 months and then annually thereafter. Radiographic assessment was conducted to detect any change in implant position, and the presence of any radiolucent lines. A radiolucent line greater than 0.5 mm was deemed significant, and the maximum width of the radiolucent line was measured in each case. Functional outcome was assessed by the Harris Hip Score (HHS).

### 2.4. Complications

Classification and diagnosis of complications in patients with metastatic disease involving the pelvis were based on Kask et al. [4]. Minor, major, and mechanical postoperative complications were reported. In the event of surgical intervention, complications were classified as major. Implant failure was categorized as mechanical complications. Implant survival was defined as the duration from the modified Harrington procedure to revision attributed to any cause, involving the acetabular or femoral component.

### 2.5. Statistical Analysis

Data analysis was performed using the Statistical Package for Social Sciences software (IBM SPSS Statistics Version 24, Chicago, IL, USA). The t-test was used for parametric values, and the Mann–Whitney U test was used for non-parametric values. The significance level was set at *p* < 0.05. The cumulative probability of remaining free of revision surgery was assessed with a Kaplan–Meier analysis.

## 3. Results

### 3.1. Functional Analysis

Functional outcome data (HHS) were collected in 12/12 patients. The average FU was 15 ± 11 (range, 6 to 36). The overall patient survival was 67% at 1 year, and 44% at 2 years. We observed DOD (died of disease) in 5/12 patients. Implant survivorship was 100% throughout the entire observation period. Further information is provided in Table 1.

The average HHS score improved from 37 ± 22 (range, 18 to 87) preoperatively to 75 ± 15 (range, 53 to 93) at the last follow-up. All patients noted a reduction in pain; however, 9/12 needed either non-opioid or opioid analgesics due to the advancement of their medical condition. All patients achieved full weight-bearing (Table 1 and Table 2).

### 3.2. Radiation Therapy

Overall, preoperative radiation was administered to 3 out of 12 patients, while 8 out of 12 patients received postoperative radiation. One patient with renal cell carcinoma did not undergo radiation therapy.

### 3.3. Implant-Related Findings

The average screw length of the 8 mm screw was 90 ± 12 mm (range, 70 to 100). Including all 6.5 mm additional fixation screws, the cumulative screw length was 161 ± 56 mm (range, 100 to 290). The median diameter of the acetabular component was 56 mm ± 4 (range, 52 to 68) (Table 1).

### 3.4. Complications

**Figure 2:**
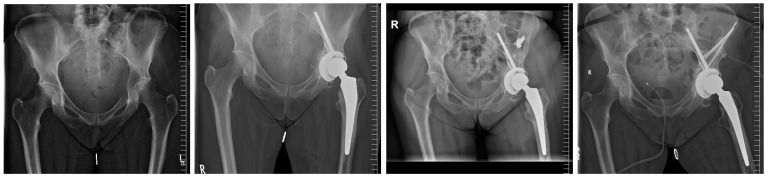
Modified Harrington Procedure for Metastatic Peri-Acetabular Bone Destruction Lesion using MUTARS^®^ PRS^®^ (Pelvic Revision Shell; Implantcast GmbH, Buxtehude, Germany)—from left to right: initial hospitalization for an acetabular metastatic lesion; modified Harrington Procedure using the PRS^®^; local progression with inadequate curettage and bone cement application ex domo; readmission for extensive curettage and k-wire reinforcement following the original Harrington concept.

There were no revisions involving the acetabular or femoral components, and neither implant loosening nor dislocation was observed. Furthermore, no instances of infection, decubitus, or hematoma were reported. In one patient, there was a local progression of the tumor involving the pelvis; however, revision surgery was performed using a different approach and did not involve implant components (Figure 2). Another patient exhibited transient nerve palsy.

In 2 out of 12 patients, we observed impaired soft tissue in the immediate postoperative period. This was primarily due to complex wound conditions in obese patients and manifested as minor wound-healing problems. In the context of declining levels of white blood cell (WBC) count and c-reactive protein (CRP) and consolidation of the surrounding wound conditions without any irritation (intraoperative specimen was negative), periprosthetic joint infection was deemed highly unlikely. A localized superficial revision without incision of the fascia was necessary for one of these patients and therefore classified as a major complication. The second patient was treated conservatively (Table 3). 

## 4. Discussion

Severe pain and immobilization are commonly encountered in metastatic destruction of the acetabulum and the peri-acetabular region. In patients with insufficient improvement under non-operative therapeutic options, surgical intervention must be considered with the main objective of attaining a stable reconstruction allowing for immediate full weight-bearing and relief of pain.

To achieve functional restoration, complex reconstruction of the pelvis is frequently required. Historically, the Harrington procedure is one of the most common treatment strategies [2,6]. Over the past decades since 1981, the Harrington procedure was modified by using screws instead of pins, the acetabulum was further supported by restoration reinforcement rings, and treatment was based on autograft and allograft or bone substitute prosthesis composite reconstruction [3,4,5,7]. Various other techniques have been reported, including antiprotrusio cages, hemipelvis endoprostheses, or saddle prostheses [7,8,9,10]. Although these types of reconstruction have provided excellent short-term outcomes, with the absence of biological fixation through osseos ingrowth, concerns persist regarding implant loosening with longer follow-ups, while the prognosis of patients with metastatic bone lesions has improved in recent years [11].

Subsequently, the importance of biological fixation may become more significant in long-term follow-ups, especially in non-radiated patients. Highly porous acetabular components have demonstrated promising results in patients with compromised bone during revision total hip arthroplasty [12,13,14,15,16,17], including the settings of metastatic lesions allowing for osseous ingrowth, even in the context of irradiated bone [18,19,20]. Overall, radiation was administered in 11 out of 12 patients under observation. While osteo ingrowth in the contact areas between bone and highly porous surface is challenging to assess, we have not documented any instances of implant loosening within our cohort.

In this series, we utilized the advantages of both fixation philosophies based on a novel generation of highly porous titanium revision shells—PRSs^®^. Contact with the host bone was accomplished by using an acetabular component with a median diameter of 56 mm ± 4 (range, 52 to 68) ± augments allowing for osseous ingrowth. In instances of lacunar defects, the conventional approach was to utilize cement augmentation, resembling a Harrington-type reconstruction. The highly porous construct provides an excellent surface for a robust interlock between the prosthesis2cement–bone interface. In addition, the construct is supported by an 8 mm dome screw, transmitting the weight-bearing load to the intact aspect of the pelvis, following the original idea of Harrington. Further reinforcement is ensured by 6.5 mm fixation screws.

The advantage of PRS over a stemmed acetabular cup (SAC) lies in the ability to freely adjust the angulation of the 8 mm dome screw within 20°, the sophisticated modular off-the-shelf (OTS) system, and the highly porous surface texture, as opposed to the microporous or smooth surface of conventional SACs [21], which have been subject of controversial discussion in the literature [22]. In the literature, reinforcement rings are considered a viable solution for addressing metastatic defects of the (peri-)acetabulum. However, unlike PRS, they do not provide authentic biological fixation, and the reconstruction of the center of rotation can present challenges [22]. Furthermore, soft tissue preparation, especially in the supraacetabular gluteal muscle region, is less invasive with PRS, as there is no need for flange or plate use. Thus, we conclude that the advantages of PRS surpass those of other established systems, a conclusion that warrants confirmation through further studies, including multicenter trials.

**Figure 3:**
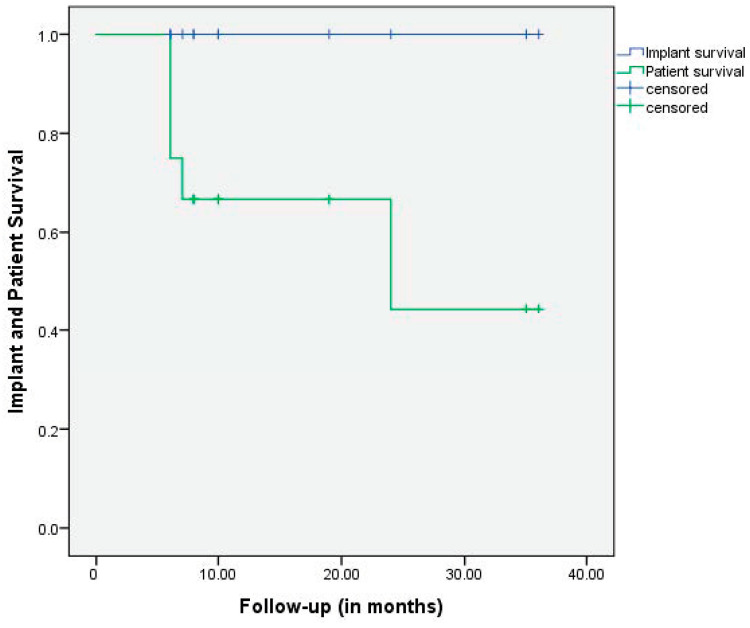
Patient survival and survivorship of the implant with further revision as the endpoint. The overall patient survival was 67% at 1 year and 44% at 2 years.

All patients achieved immediate full weight-bearing, providing sufficient and reliable improvement in pain and function. The average HHS score improved from 37 ± 22 (range, 18 to 87) preoperatively to 75 ± 15 (range, 53 to 93) at the last follow-up. Khan et al. reported comparable values with a mean of 74 postoperatively in a similar cohort [18]. Revision-free survival of PRS^®^ was 100% at 1 yr and 2 yrs (Figure 3).

Given the limited number of patients overall and the relatively short follow-up duration, statements about survival probabilities must be approached with caution in the context of statistical significance.

Radiographic evaluation revealed well-fixed implants with no signs of aseptic loosening or implant movement at the last follow-up. We would like to emphasize that the complex patient cohort is facing massive acetabular defects, underwent radiation, and was commonly treated with chemotherapy and/or immunosuppressive treatment algorithms perioperatively.

While advanced oncological treatment contributes to the prolonged survival of some patients, we observed a 33% mortality rate due to disease (dead of disease, DOD) within the first year following surgery and 56% DOD at 2 years (Figure 3). Our patient survival rate is largely in accordance with the literature, with 1-year survival ranging between 42% and 49% [6,23,24].

Overall, complications were observed in 5 out of 12 (41%) patients, with only 2 requiring revision surgery, excluding the involvement of implant components (Table 3). Our findings are similar to other authors; complications in this complex field of surgery are frequently seen [5,7]. Detailed analysis of complication rates is challenging, given the variety of techniques employed to address metastatic diseases, the lack of standardized reporting of complications, and the tendency to underreport complications.

We have demonstrated immediate full weight-bearing, even in large peri-acetabular defects, with no signs of loosening, framed by an acceptable rate of complication. The highly porous surface ensures optimal primary stability—particularly when combined with bone cement, while secondary osteo ingrowth in the context of sufficient systemic treatment of the tumor is still feasible, in contrast to the frequently used Burch–Schneider cage. The presented technique is easy to combine with K-wire or Steinmann pin augmentation from the iliac crest (Figure 2); however, this is frequently not necessary, highlighting the exceptional primary stability of the 8 mm dome screw and the acetabular component.

### Limitations

This is the first study to report on the clinical and radiological outcomes of this novel implant in patients with metastatic destruction of the acetabulum and the peri-acetabular region. Although the data were prospectively collected, the patients were reviewed retrospectively and we consider that prospective, controlled, and randomized studies would be preferable. This study was limited to a single institution. In addition, the cohort size was limited, consistent with the rarity of the disease under consideration and the just recently introduced implant system PRS^®^.

Despite the short-term nature of the follow-up, the study design sufficiently addresses the question of effective management in cases of metastatic destruction of the peri-acetabulum and the feasibility of immediate full weight-bearing.

## 5. Conclusions

PRS^®^ enables immediate full weight-bearing due to its surface characteristics and biomechanical properties, while the 8 mm dome screw with a length of up to 10 cm efficiently transmits the weight-bearing load to the intact aspect of the pelvis. To summarize, this technique is effective, reproducible, and exhibits excellent implant survival in patients with destructive metastatic lesions of the acetabulum and the peri-acetabular region. Furthermore, it is distinguished by a satisfactory surgical duration and relatively low rates of complications.

## Figures and Tables

**Table 1:** Demographics and characteristics.

	PRS^® a^
*n*	12
Enrollment period	2020–2023
Follow-up ^b^ (in months)	15 ± 11 [6–36]
Age ^b^ (in years)	70 ± 8 [56–79]
Women (%)	7 (58)
BMI ^b^	26 ± 4 [19–34]
ASA score ^c^	3 ± 1 [2–4]
Right joint; *n* (%)	8 (67)
DOD ^d^	5/12
HHS preoperatively ^e^	37 ± 22 [18–87]
HHS at latest follow-up ^e^	75 ± 15 [53–93]
**Implant related findings**	
Overall PRS implant size ^b^ (mm)	56 ± 4 [52–68]
Tripolar acetabular systems; n (%)	6 (50)
->PRS^® a^ implant size ^b^ (mm)	60 ± 4 [56–68]
Cemented PE-Cup; n (%)	6 (50)
->PRS^® a^ implant size ^b^ (mm)	54 ± 2 [52–56]
Modular metal augments; n (%)	2 (17)
Number of additional fixation screws ^b^	3 ± 1 [2–7]
Added screw lengths (mm) ^b^	161 ± 56 [100–290]
Lengths of 8 mm screw (mm) ^b^	90 ± 10 [70–100]
Added lengths of solely 6 mm screws (mm) ^b^	71 ± 56 [0–100]
Duration of surgery (minutes) ^b^	172 ± 34 [118–220]

^a^ PRS^®^ (**P**elvic **R**evision **S**hell—Implantcast GmbH, Buxtehude, Germany) ^b^ in ‘mean + SD [range]’; ^c^ in ‘median ± SD [range]; ^d^ died of disease; ^e^ significant improvement of the HHS (*p*-value: **0.000**).

**Table 2:** Series of 12 consecutive patients with acetabular and peri-acetabular metastases were treated with the modified Harrington procedure using PRS^®^.

Patient Number	Age (Years)	Primary Tumor	ASA ^a^	Mobilization Preoperative	Mobilization at Latest FU ^b^	Follow-Up (Months)	Patient Survival	Implant Survival (Yes/No)
#1	70	Multiple myeloma	3	Two crutches	Unaided	24	DOD ^c^	yes
#2	59	Prostate cancer	4	Wheelchair	Cane	7	DOD ^c^	yes
#3	76	Breast cancer	3	Wheelchair	Two crutches	6	DOD ^c^	yes
#4	73	Non-Hodgkin lymphoma	4	Wheelchair	Cane	35	AWD ^d^	yes
#5	82	Breast cancer	3	Wheelchair	Two crutches	6	DOD ^c^	yes
#6	74	Breast cancer	3	Wheelchair	Unaided	19	AWD ^d^	yes
#7	69	Renal cell carcinoma	3	Two crutches	Two crutches	6	DOD ^c^	yes
#8	56	Bronchial cancer	4	Wheelchair	Two crutches	10	AWD ^d^	yes
#9	60	Bronchial cancer	4	Wheelchair	Walking frame	8	AWD ^d^	yes
#10	79	Prostate cancer	3	Two crutches	Cane	10	AWD ^d^	yes
#11	66	Breast cancer	3	Two crutches	Cane	8	AWD ^d^	yes
#12	75	Breast cancer	2	Wheelchair	Cane	36	AWD ^d^	yes

^a^ ASA: American Society of Anesthesiologists; ^b^ FU: follow-up; ^c^ DOD: died of disease; ^d^ AWD: alive with disease.

**Table 3:** Complications of the modified Harrington procedure using PRS^®^.

Complications	Mechanical Failure	Dislocation	Local Progression of Tumor	Infection	Decubitus	Hematoma	Nerve Injuries	Impaired Soft Tissue	Ʃ
Major	0	0	1 ^a^	0	0	0	0	1	2
Minor	0	0	0	0	0	1	1	1	3
Overall			1			1	1	1	5

^a^ Revision surgery did not involve the acetabular or femoral component.

## Data Availability

The data presented in this study are available on request from the corresponding author due to privacy and legal reasons.
